# Computational prediction and experimental validation of *Salmonella* Typhimurium SopE-mediated fine-tuning of autophagy in intestinal epithelial cells

**DOI:** 10.3389/fcimb.2022.834895

**Published:** 2022-08-17

**Authors:** Amanda Demeter, Anne-Claire Jacomin, Lejla Gul, Ashleigh Lister, James Lipscombe, Rachele Invernizzi, Priscilla Branchu, Iain Macaulay, Ioannis P. Nezis, Robert A. Kingsley, Tamas Korcsmaros, Isabelle Hautefort

**Affiliations:** ^1^ Earlham Institute, Norwich Research Park, Norwich, United Kingdom; ^2^ Quadram Institute Bioscience, Norwich Research Park, Norwich, United Kingdom; ^3^ Department of Genetics, Eotvos Lorand University, Budapest, Hungary; ^4^ School of Life Sciences, University of Warwick, Coventry, United Kingdom; ^5^ School of Biological Sciences, University of East Anglia, Norwich, United Kingdom; ^6^ Faculty of Medicine, Department of Metabolism, Digestion and Reproduction, Imperial College London, London, United Kingdom

**Keywords:** *Salmonella* Typhimurium, autophagy, SopE, network biology, MAP1LC3B, Host-microbe interactions

## Abstract

Macroautophagy is a ubiquitous homeostasis and health-promoting recycling process of eukaryotic cells, targeting misfolded proteins, damaged organelles and intracellular infectious agents. Some intracellular pathogens such as *Salmonella enterica* serovar Typhimurium hijack this process during pathogenesis. Here we investigate potential protein-protein interactions between host transcription factors and secreted effector proteins of *Salmonella* and their effect on host gene transcription. A systems-level analysis identified *Salmonella* effector proteins that had the potential to affect core autophagy gene regulation. The effect of a SPI-1 effector protein, SopE, that was predicted to interact with regulatory proteins of the autophagy process, was investigated to validate our approach. We then confirmed experimentally that SopE can directly bind to SP1, a host transcription factor, which modulates the expression of the autophagy gene *MAP1LC3B*. We also revealed that SopE might have a double role in the modulation of autophagy: Following initial increase of *MAP1LC3B* transcription triggered by *Salmonella* infection, subsequent decrease in *MAP1LC3B* transcription at 6h post-infection was SopE-dependent. SopE also played a role in modulation of the autophagy flux machinery, in particular MAP1LC3B and p62 autophagy proteins, depending on the level of autophagy already taking place. Upon typical infection of epithelial cells, the autophagic flux is increased. However, when autophagy was chemically induced prior to infection, SopE dampened the autophagic flux. The same was also observed when most of the intracellular *Salmonella* cells were not associated with the SCV (strain lacking *sifA*) regardless of the autophagy induction status before infection. We demonstrated how regulatory network analysis can be used to better characterise the impact of pathogenic effector proteins, in this case, *Salmonella*. This study complements previous work in which we had demonstrated that specific pathogen effectors can affect the autophagy process through direct interaction with autophagy proteins. Here we show that effector proteins can also influence the upstream regulation of the process. Such interdisciplinary studies can increase our understanding of the infection process and point out targets important in intestinal epithelial cell defense.

## Introduction

Invasion and survival of intracellular bacterial pathogens within mammalian cells results from the timely expression of an arsenal of virulence factors, often horizontally acquired (Marcus et al., 2000; Hansen-Wester and Hensel, 2001; Nieto et al., 2016). The zoonotic *Salmonella enterica* subsp. *enterica* serovar Typhimurium pathogen (*S.* Typhimurium for short) is no exception to that rule (Srikumar et al., 2015; Colgan et al., 2016; Powers et al., 2021). Like many other enteric pathogens, *S.* Typhimurium hijacks the host cell machinery to enter the host cells, to hide from the innate immune system and ultimately to survive and spread to the next host (Alpuche Aranda et al., 1992; Lucas and Lee, 2000; Kröger et al., 2013). For that, *Salmonella* expresses and coordinates the secretion of its effectors directly into the host cell cytosol through type three secretion systems (T3SS), encoded on two *Salmonella* Pathogenicity Islands (SPIs) (Marcus et al., 2000; Schlumberger and Hardt, 2005; Lou et al., 2019).

Upon entry, *S.* Typhimurium will reside in a membrane-bound vacuole called the *Salmonella*-containing vacuole (SCV), where it may replicate. *Salmonella* entry is mediated by molecular mimics of host proteins (such as Guanine Exchange Factors (GEFs)) through effectors, including SopE, SopE2 and SopB, that activate host Rho-GTPases, RAC1, CDC42 (Schlumberger and Hardt, 2005), and the GTPase activating protein (GAP) SptP that subsequently deactivates Rho-GTPases, resolving host cell apical changes (Srikanth et al., 2011). In 20% of the cases entry into non-phagocytic cells is followed by an escape of *Salmonella* from the SCV (Brumell et al., 2002; Beuzón et al., 2002; Castanheira and García-Del Portillo, 2017). The resulting cytosolic *Salmonella* population must then adapt to the cytosol environment by mechanisms that are not fully elucidated, although SPI-1 effectors, including SopB, SptP, SipA, SopA, SopB, SopD and SopE may play additional roles impacting cytosolic *Salmonella* (Kubori and Galán, 2003; Drecktrah et al., 2005; Giacomodonato et al., 2007). Consistent with the idea that SopE could play a critical role in the adaptation of the pathogen to the host cell cytosol, SopE and SopE2 remain detectable on the SCV membrane up to 6 hours post-infection (Vonaesch et al., 2014).

Autophagy is a ubiquitous process crucial for cell homeostasis and stress survival of eukaryotic cells. Double membrane structures called autophagosomes are generated inside the cells and engulf superfluous organelles and proteins as well as invading pathogens. Autophagosomes then fuse with lysosomes, leading to degradation of the content (Feng et al., 2014; Cicchini et al., 2015; Sorbara and Girardin, 2015). By eliminating intracellular pathogens, such as bacteria and viruses (Sorbara and Girardin, 2015), autophagy assists the immune system in fighting infectious agents.

Over 38 proteins are involved in the autophagy process, each being temporally regulated throughout the different stages of the process: initiation, cargo recognition by the ATG ubiquitination system, membrane nucleation permitting the double membrane autophagosome formation, maturation of the compartment and fusion with the lysosome (Quan and Lee, 2013; Cicchini et al., 2015; Türei et al., 2015). We have previously described a computational pipeline allowing identification of interactions between secreted effector proteins of bacterial pathogens and autophagy core proteins (Sudhakar et al., 2019). In another previous study, we had generated and manually curated an autophagy protein interaction network in which we grouped the core autophagy proteins based on the following phases of autophagy: induction; cargo recognition and packaging; Atg protein cycling; vesicle nucleation; vesicle expansion and completion; transport of autophagosome; fusion with the lysosome (Kubisch et al., 2013).

Among those proteins, the MAP1LC3B (LC3-II) receptor, a ubiquitin-like protein, plays a critical role in the capture of the cargo into the autophagosome. When the autophagy process is activated, MAP1LC3B gets lipidated and associated with the double membrane of the autophagosome. In concert with autophagy adaptors (e.g. p62, NDP52, OPTN), MAP1LC3B binds to the targeted protein, ensuring its capture for lysosomal degradation (Shaid et al., 2013; Khaminets et al., 2016).

Although autophagy is a robust clearing process against intracellular pathogens, some infectious agents, including *S.* Typhimurium have developed ways to escape or hijack autophagy for their own benefit. *S.* Typhimurium can subvert host autophagy at several stages of this process (Baxt et al., 2013; Baxt and Xavier, 2015; Sorbara et al., 2018). *Salmonella* secretes several effectors that have been proposed to interact with the ubiquitin pathway, such as the E3 ligases SopA, SspH1, SspH2 and SlrP, and the deubiquitinases SseL and AvrA (Baxt et al., 2013; Herhaus and Dikic, 2018). SopB can prevent fusion of the autophagosome with the lysosome (Weigele and Alto, 2010). Furthermore, cytosolic *S.* Typhimurium interacts with autophagy proteins, particularly MAP1LC3B and p62 proteins (Yu et al., 2014). Autophagy can also promote bacterial growth by sealing damaged SCVs maintaining a suitable environment for replication (Kreibich et al., 2015). It is therefore apparent that *Salmonella*-mediated modulation of autophagy is a more complicated and dynamic process that remains to be fully elucidated.

In this study, we present a network approach and describe its application to predict interactions of *Salmonella* effectors with host transcription factors, potentially resulting in changes in expression of key autophagy genes. We experimentally validate this prediction for the GEF-mimicking effector SopE and show that it can also modulate the flux of autophagy later on during infection.

## Materials and methods

### Computational predictions of interactions between Salmonella effectors and core autophagy genes

Protein-protein interactions (PPIs) were inferred by domain-domain interaction (DDI) prediction using the MicrobioLink pipeline (Andrighetti et al., 2020). Briefly, DDIs with high confidence values (interactions predicted by at least two different *in silico* methods or using multiple sources) were collected from the DOMINE resource (Yellaboina et al., 2011). We assumed that *Salmonella* and human proteins are connected if the interacting domains were represented in the database. The interaction prediction was merged with already existing predictions (Krishnadev and Srinivasan, 2011; Kshirsagar et al., 2012) and experimentally validated transcription factor-gene interactions related to autophagy.

Scripts for processing the interaction predictions, databases and other tables were written in R (See **Table S1** for a list of host transcription factors and bacterial effectors found to interact). For the network analysis the Cytoscape network visualization program was used (Shannon et al., 2003). Autophagy core genes and their direct transcriptional interactions were downloaded from the following databases: Autophagy Regulatory Network (ARN, http://autophagyregulation.org/, (Türei et al., 2015), HTRIdb (http://www.lbbc.ibb.unesp.br/htri, (Bovolenta et al., 2012) and TRRUST (https://www.grnpedia.org/trrust/, (Han et al., 2015). All scripts are available at https://github.com/korcsmarosgroup/HMIpipeline.

### SopE-SP1 immunoprecipitation assay

HEK293 cells grown to 50% confluence were transfected with plasmids for overexpression of BirA-Myc-SP1 (gift from Markku Varjosalo; Addgene plasmid #167726) and GFP or GFP-SopE (Yuki et al., 2019) using GeneJuice transfection reagent according to the manufacturer’s recommendations. Protein extracts were recovered 24-hour post-transfection in lysis buffer (20mM Tris ph7.5, 0.5% Triton-X100, 150mM NaCl, 2mM EDTA) supplemented with EDTA-free proteases inhibitors cocktail (04693132001, Roche) and benzonase (1.03773.1010, Millipore). Co-immunoprecipitations were performed on cleared lysates with GFP-Trap Magnetic beads (gtd, Chromotek) overnight at 4°C. Four consecutive washes with the lysis buffer were performed before the bead suspension was added in sample loading buffer (Sigma) and incubated for 5-10 min at 95°C. Inputs and IP samples were loaded onto 4-20% polyacrylamide gels and were transferred onto PVDF membranes (cold wet transfer in 10% ethanol for 1h at 100V). Membranes were blocked in 5% non-fat milk in TBST (0.1% Tween-20 in TBS) for 1 h. Primary antibodies anti-Myc (Cell Signaling #2276) and anti-GFP (sc-9996, Santa Cruz Biotechnology) diluted in TBST were incubated overnight at 4°C with gentle agitation. HRP-coupled secondary antibodies binding was done at room temperature (RT) for 45 min in 1% non-fat milk dissolved in TBST. All washes of the membranes were performed for 10 min in TBST at RT. Probed membranes were developed using a Chemidoc imaging system (BioRad) and signal intensity quantification was performed by measuring the densitometry of appropriate bands on not overexposed membranes using FiJi/ImageJ.

### Bacterial strains and culture conditions

All bacterial strains are listed below in **Table 1**. Gene deletion mutant strains were developed from the JH3009 (here referred to as wild type, wt) strain. The *sopE* gene was replaced in *S.* Typhimurium with the *aphII* gene conferring resistance to kanamycin, using a method LambdaRed recombination method (Datsenko and Wanner, 2000), with the exception that the red recombinase was supplied on the pSIM18 plasmid (Chan et al., 2007). The *aphII* gene from pKD4 was amplified using oligonucleotide primers 5’- CCTGCTATCTATATATAAATGAATTATGTACATATAAAAGGATCATTACCgtgtaggctggagctgcttcg-3’ and 5’- GGTTCATATTAATCAGGAAGAGGCTCCGCATATTTTTTGGTTTTTCAGTGTcatatgaatatcctccttagt-3’ and introduced into strain SL1344 containing the pSIM18 plasmid. Recombinant transformants were selected on LB agar plates containing 50 µg ml-1 kanamycin. The *sopE* gene deletion was reintroduced into SL1344 by P22 transduction to minimise off-target mutations, as described previously (Kingsley et al., 1999). The genotype was confirmed by PCR amplification across the *sopE* locus using oligonucleotides 5’-CAGATGGACATAGCATTTGC-3’ and 5’-ATGACGGTTTAGCTCCGGAG-3’.

**Table 1:** *Salmonella enterica* Typhimurium strains, generated and used in this study.

Strain	Description	Reference
**S**L1344	4/74 *hisG rpsL*	Hoiseth and Stocker, 1981
JH3009 (named wt in this manuscript)	SL1344, ɸ (*ssaG*’-*gfp^+^*), Cm^R^	Hautefort et al., 2003
SL1344Δ*sopE*	SL1344Δ*sopE*, Km^R^	This study
TK0014	JH3009 ɸ(*ssaG’-gfp^+^*), Δ*sopE*, Km^R^, Cm^R^	This study
TK0019	JH3009 ɸ(*ssaG’-gfp^+^*), Δ*sifA*, Km^R^, Cm^R^	Sudhakar et al., 2019
TK0021	SL1344, Δ*sifA*, Km^R^	Sudhakar et al., 2019
TK0026	JH3009 ɸ(*ssaG’-gfp^+^*), Δ*sifA* Δ*sopE*, Km^R^, Cm^R^	This study

Combination of the ɸ(*ssaG’-gfp^+^*) intracellular reporter fusion with both the *sifA* and *sopE* gene deletions was carried out as follows in the SL1344 genetic background. The Kanamycin resistance cassette replacing the *sifA* gene in TK0021 was excised from the chromosome using the yeast Flp recombinase expressed from the thermosensitive replicon pCP20. The pCP20 replicon was subsequently removed from TK0021 after culture at 40°C in non-selective medium. The *sopE*, Km^R^ deletion and the ɸ(*ssaG’-gfp^+^*), Cm^R^ transcriptional fusion were transduced into the *sifA*, Km^S^ new strain by P22 phage transduction, generating the strain TK0026 used in this study (See **Table 1**) (Gemski and Stocker, 1967). The generated the strain TK0026, lacking both SifA and SopE and carrying a GFP reporter system to indicate intracellular *Salmonella* location in infected epithelial cells.

Bacterial strains were grown in 5 ml of LB broth overnight (Sambrook and Russell, 2001) at 37°C at 250 rpm. For invasion assays, a 1:100 dilution of the overnight bacterial culture was grown in 25 ml of LBS (LB containing a total of 0.3 M NaCl) in 250 ml conical flasks until an optical density of 1.2 was obtained at 600 nm (A600). Antibiotics were added as required at the following final concentrations (kanamycin, 50 mg ml-1; chloramphenicol, 10 mg ml-1).

### HT-29 cell culture and invasion assays

HT-29 human colon cancer epithelial line (HTB-38, ATCC) was cultured in DMEM supplemented with 10% heat-inactivated Fetal Bovine Serum (FBS) and 2 mM L-Glutamine at 37°C, 5% CO_2_. HT-29 epithelial cells were seeded into 6- and 24-well cell culture plates at a density of 3.2x10^6^ and 2x10^5^ cells/well, respectively, to obtain 80% confluency by the day of the invasion assays (48h later). Six well-plates were used to generate enough infected epithelial cells for RNA extraction and qPCR autophagy gene expression analysis. Twenty four-well plates were used for immunofluorescence microscopy monitoring of autophagy flux upon infection. In those plates, HT-29 cells were seeded on 13mm diameter glass round coverslips. Three biological replicates were used for each condition and time point. Where necessary, autophagy was induced by treating the cells with 30 µg ml-1 rapamycin or DMSO only prior to and during the 6h long infection (17h total). On the day of the invasion assay, cells were washed twice in non-supplemented DMEM followed by the infection. Bacterial suspensions were prepared in DMEM from the LBS sub-culture of the *Salmonella* strains (see Bacterial strains and culture conditions) at a multiplicity of infection (MOI) of 10 bacterial cells per mammalian cell. Infected cells were incubated for 30 minutes at 37°C, 5% CO_2_. The infection medium was then replaced with a complete medium containing 30 µg ml-1 gentamicin for 30 minutes to kill the remaining extracellular *Salmonella* cells. For the rest of the experiment, the gentamicin concentration was then reduced to and maintained at 5 µg ml-1.

For the assay allocated to autophagy flux bioimaging, the medium was removed at 6h post infection (p.i.). Cells were washed twice in Dubelcco’s phosphate-buffered saline (DPBS; D8537, Sigma Aldrich), fixed in 4% paraformaldehyde at room temperature for 20 min, and washed twice for 5 min at room temperature in DPBS prior to immunofluorescence labelling.

### Cell sorting

At 2h and 6h post-infection, the medium was removed and cells were washed twice in DPBS. Each sample well was trypsinised in 50% Trypsin-Versene (EDTA), 49.6% DPBS, 2mM EDTA and 5 µg ml-1 gentamicin for 5 minutes at 37°C. The trypsin reaction was stopped in 89.6% DPBS, 10% FBS, 2mM EDTA and 5 µg ml-1 gentamicin (i.e. FACS buffer). Single-cell suspensions were obtained by pipetting several times. Cells were washed and resuspended in FACS buffer. Infected cells and bystanders were separated by Fluorescence-Activated Cell Sorting on a BD FACSMelody machine (BD Biosciences). Sorting gates were set based on negative cells (from an uninfected well) and positive control cells (from an infected well) based on the level of GFP present in each cell. Eight pools of 50 epithelial cells were sorted from each condition and time point (4 x GFP- as non-infected cells or “bystanders’’ and 4 x GFP+ as “*Salmonella*-containing epithelial cells”) into 96-well plates containing 2 μl of lysis buffer (0.2% Triton X-100 and 2 U µl-1 RNase inhibitor). Samples were then processed as detailed in the RNA samples extraction and processing section.

### RNA samples extraction and processing

The low input RNA extraction and reverse transcription were carried out following the SmartSeq2 protocol previously described (Picelli et al., 2013). Reverse transcription was performed as in the following steps. First, Oligo-dT30VN primer was added to the sample lysates at 2.5 µM, 2.5 nM each dNTP final, and priming reactions were incubated at 72°C for 3 mins. First strand synthesis was subsequently initiated by addition of 1.03 µM custom template switching oligo, 6.18 µM MgCl2, 1.03 M Betaine, 5.12 mM DTT, 1.03x Superscript First Strand Buffer, 0.52 U µl-1 RNase Inhibitor, 10.3 U µl-1 SuperScript II reverse transcriptase in nuclease-free water. The reverse transcription reactions followed the successive steps: 42°C 90 mins, 10×(50°C 2 mins, 42°C 2 mins), and 70°C 15 mins. Finally, 15 ul PCR mastermix (1.6x KAPA HiFi Hotstart Readymix, KAPABIOSYSTEMS, 0.16 µM IS primers, nuclease-free water) was added to each sample. PCR cycles were as follows, 98°C 3 mins, 21×(98°C 20 secs, 67°C 15 secs, 72°C 6 mins), 72°C 5 mins). PCR products were cleaned up with 0.8x volumes of Ampure XP and 80% ethanol. Samples were then eluted in 20 µl 10 mM Tris-HCl.

### qPCR

Taqman gene expression analysis was conducted on the SmartSeq2 amplified cDNA. In brief, TaqMan™ Fast Advanced Master Mix (4444557, ThermoFisher Scientific) was used for all qPCR reactions, assay probes and samples were dispensed into 384-well Roche-style qPCR plates (4titude, 4ti-1381) using a Mosquito HV automated liquid handling instrument (SPT Labtech), to a final reaction volume of 1.6µl (80nl 20× TaqMan^®^ Gene Expression Assay, 800nl 2× TaqMan^®^ Gene Expression Master Mix, 720nl normalised cDNA). Samples were then analysed on a Roche Lightcycler 480. The Taqman gene expression assay probes were used for the *MAP1LC3B* test gene and the beta-2-microglobulin *B2M* reference gene (Hs00797944_s1 4453320 and Hs00984230_m1 4453320, respectively, ThermoFisher Scientific).

Gene expression was normalised to the B2M internal reference gene and plotted as log2^-deltaC_T_ (Rao et al., 2013). First, technical replicates with the smallest and largest C_T_ values (raw C_T_ in case of potential reference gene and normalised C_T_ values in case of genes of interest) were excluded for each gene within each condition.

### Immunohistochemistry

MAP1LC3 and p62 were labelled as previously done (Sudhakar et al., 2019). In brief, for MAP1LC3B/LC3B immunostaining, cells were quenched at room temperature in 50 mM NH4Cl in DPBS for 10 min, then permeabilized in methanol for 5 min and washed in DPBS 3 times for 5 min. This was followed by blocking the samples at room temperature in 1% bovine serum albumin (BSA) Fraction V (05479, Sigma-Aldrich) in DPBS for 30 min. The rabbit anti-MAP1LC3B/LC3B (ab48394, Abcam) antibody was applied overnight at 4°C. The antibody labelling solution was diluted at a 1:2000 dilution in DPBS containing 1% BSA fraction V (05479, Sigma Aldrich). SQSTM1/p62 immunolabeling was performed as follows. Fixed cells were permeabilized and blocked in a solution containing 1% BSA and 0.1% saponin (84-510, Fluka), in DPBS for 30 min at room temperature. The rabbit anti-SQSTM1/p62 antibody (ab91526, Abcam) was applied overnight at 4°C at a f 1:6000 dilution in DPBS containing 1% BSA and 0.1% Saponin.

The FITC-conjugated anti-GFP antibody (ab6662, Abcam) was applied overnight at 4°C in all samples at a 1:200 dilution in all primary solutions.

All primary antibodies were washed 3 times in either 1% BSA in DPBS (MAP1LC3B/LC3B) or 1% BSA and 0.1% saponin in DPBS (SQSTM1/p62). Alexa Fluor 594-conjugated anti-rabbit secondary IgG (ab91526, Abcam) was diluted 1 in 1000 and applied to all samples at room temperature for 1 h. All samples were counterstained with DAPI at a dilution of 1:2000 in the buffer respective to each primary antibody. Samples were then washed 3 times in their respective buffers, once in water and finally mounted on microscopy glass slides. Coverslips were mounted in Aqua-poly/mount anti-fading compound (18606, Polysciences Inc.). Coverslips were left to set, sealed using nail varnish and stored at -20°C until observation. Slides were imaged on a Zeiss LSM710 microscope, using a 100x Apochromat (100x/1.4 Oil DIC plan Apo) oil immersion objective. Focal plan and laser power/gain was kept constant throughout the acquisition process. Over 500 epithelial cells were imaged per condition tested. The acquisition was semi-blind with conditions revealed post-analysis.

### Image analysis

The analysis of SQSTM1/p62 and LC3 staining was done using semi-automated macros within FiJi (Image J2) software. To avoid unconscious-bias, imaged areas were chosen randomly based solely on the DAPI staining. DAPI staining was used for the identification of nuclei and individual cells were identified by extension of the nuclei mask. Segmented cells touching the edge of the images or artefacts (small objects) were eliminated. Individual regions of interest (ROIs) were saved for each image and subsequently used for quantification of SQSTM1/p62 and LC3 staining. Intensity and puncta information for individual cells in each fluorescent channel was exported into Excel spreadsheet and used for statistical analysis.

### Statistical tests

Distribution normality of data points was determined by the Shapiro-Wilk test and the equality of variance was determined by the Levene’s test. To compare 3 or more groups, one-way ANOVA (for normal distribution) or Kruskal-Wallis (for non-normal distribution) tests were performed using R. On statistically different samples the appropriate *post hoc* test was applied: Tukey following ANOVA and kruskalmc following Kruskal-Wallis. T-tests and kruskalmc tests were performed to compare LC3 and p62 protein levels in wild type *Salmonella*-infected cells and cells infected by the Δ*sopE* deletion strain derivative.

## Results

### Network analysis of potential pathogen-host interactions affecting autophagy

The potential effect of *S.* Typhimurium effector proteins on autophagy gene expression was analysed using a network of predicted and experimentally validated interactions. We predicted pathogen-host interactions based on known domain-domain interactions and supplemented this with two predictions previously described (Krishnadev and Srinivasan, 2011; Kshirsagar et al., 2012) making up our merged list of interactions (See **Table S1**). The source proteins were filtered for secreted *S.* Typhimurium effectors (Miao and Miller, 2000; Ehrbar and Hardt, 2005; Haraga et al., 2008; Figueira et al., 2013), and the target proteins were filtered for those that had previously been validated experimentally as transcription factors of autophagy-core genes (Bovolenta et al., 2012; Han et al., 2015; Türei et al., 2015). The core autophagy network was extended with an additional gene, *ATG4D* and its connection to *GABARAPL1* (Betin and Lane, 2009). As further filtering steps, transcription factors were kept if they were predicted to connect to only one or few *Salmonella* effectors (eliminating some potential false positives) while at the same time regulating more than one of the core autophagy genes.

The first layer of the constructed network was centred around the interactions between *S*. Typhimurium virulence proteins (excluding SlrP) and human autophagy core-regulating transcription factors. Regarding these interactions there was no overlap between the three sources, and only three interactions of SlrP with CTCF, YY1 and SP1 were overlapping between the prediction of Kshirsagar et al. and our prediction. This layer of the final network contains 71 connections from Kshirsagar, 0 connections from Krishnadev and 4 connections from our prediction (Krishnadev and Srinivasan, 2011; Kshirsagar et al., 2012). On the human side there were 154 transcription factor-autophagy core interactions from ARN, 105 from HTRI and TRRUST database, with altogether 33 transcription factors, 35 core autophagy genes and 159 regulatory interactions between them.

**Figure 1:**
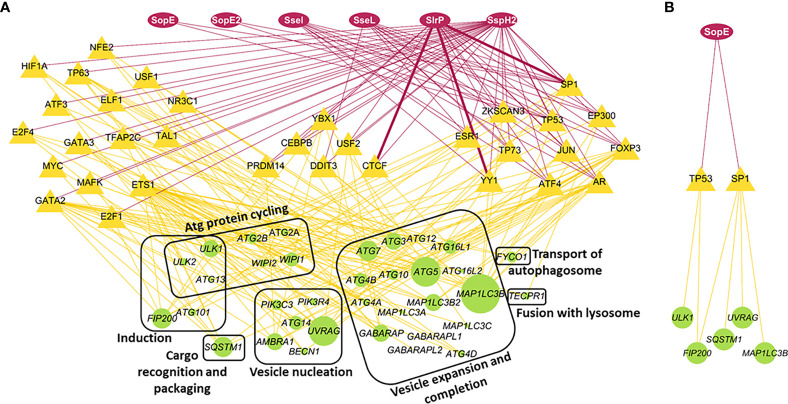
Interaction network between selected *Salmonella* Typhimurium effectors, host cell transcription factors and autophagy core genes. **(A)** Network analysis of potential interactions between *Salmonella* and host autophagy. Red ovoid nodes are the selected *Salmonella* effector proteins. Red edges are PPI predictions. Thin edges were predicted by one of the three methods. Thick edges were predicted by two of the three methods. Yellow triangular nodes are host transcription factors that *Salmonella* effectors can influence. These are clustered according to the number of *Salmonella* effectors they are targeted by. Yellow edges reflect transcriptional regulation of core autophagy genes (round green nodes) by the transcription factors. The size of the green nodes is proportional to the number of transcription factors they are connected to. **(B)** Subnetwork illustrating the potential interaction of the *Salmonella* effector SopE with transcription factors affecting specific autophagy core genes. The same layout was used here as for the large network.

Our network analysis highlights that several *Salmonella* effectors can impact several transcription factors. For example, effectors such as SseI, SseL, SlrP and SspH2 target 9, 10, 16 and 33 transcription factors, respectively (**Figure 1A**). This reflects the multiple routes *Salmonella* has evolved to subvert host intracellular clearance mechanisms, here by affecting all stages of the autophagy process (from induction, through to the autophagosome formation to the fusion with lysosomes; **Figure 1**). We selected six *S*. Typhimurium proteins that were not predicted to connect to too many of the human transcription factors (except SspH2) to simplify our further analysis. One of these selected effectors is SlrP. SlrP, that can be secreted through the SPI-1 and -2 apparatus as well, and is an E3 ubiquitin ligase, hence potentially able to interact with ubiquitin-mediated autophagy. Moreover, it has overlapping predicted interactions in two of the PPI predictions, highlighting the power of our computational approach. Two additional effectors, SopE and SopE2, are SPI-1 effectors and mimic host GEFs. They are particularly important in the membrane ruffling associated with *Salmonella* entry into the non-phagocytic epithelial cells. Yet, they are also expressed in later stages of the invasion and present in our network possible interactions with two autophagy transcription factors. The remaining of the selected proteins, SseI, SseL and SspH2 are SPI-2 virulence effectors, which means that they are probably secreted in the later stages of invasion and SseL and SspH2 are also among the proteins that can alter ubiquitination.

Considering SopE’s role in altering RhoGTPases downstream effect and persisting long enough in the host cells to be exposed to the host cell machinery, including the autophagy process (Schlumberger and Hardt, 2005; Vonaesch et al., 2014), it was subsequently selected for experimental validation of our predictions. When focusing on the SopE potential interactions with autophagy regulators, we predicted that SopE can interact with only two key transcription factors, TP53 regulating genes in the autophagy induction phase, and SP1, controlling the formation and expansion of the autophagosome compartment (**Figure 1B**). These two steps of autophagy are likely to be influenced differently depending on the location of the intracellular *Salmonella* cells, either within a damaged SCV or cytosolic. We investigated further if the GEF-mimicking SopE influenced the autophagy process at different levels, focusing only on the SopE and SP1 potential interaction.

### SopE can directly interact with the host SP1 transcription factor

**Figure 2:**
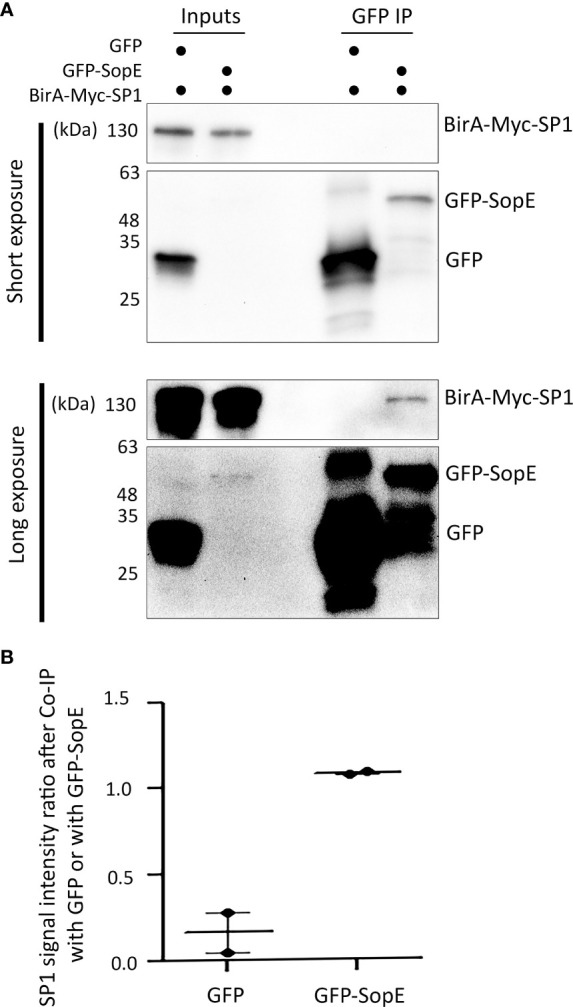
The *Salmonella* effector SopE can bind to the human transcription factor SP1, as predicted. **(A)** Co-immunoprecipitation of GFP-SopE and BirA-Myc-SP1 ectopically expressed in HEK293 cells. **(B)** SP1 signal intensity ratio after Co-IP with GFP or with GFP-SopE showing enrichment of SP1 when GFP-SopE is used compared with GFP alone, based on two independent measurements.

Our network analysis suggests that one way *Salmonella* can modulate autophagy flux is through altering the regulation of autophagy-related gene expression. For example, we predicted that SopE can interact with SP1 and TP53 (**Figure 1B**). Our study focuses later on the modulation of the autophagy process through MAP1LC3B, so we centred our experimental validation on the potential SopE-SP1 interaction. To validate this hypothesis, we performed a co-immunoprecipitation assay between GFP-SopE and BirA-Myc-SP1 recombinant proteins, ectopically expressed in HEK293 cells (**Figure 2A**). We observed that SP1 is enriched over sixfold with GFP-SopE compared to GFP alone (**Figure 2B**), confirming the direct interaction of the *Salmonella* SopE protein with the SP1 autophagy regulator. This suggests that through interaction with SP1, SopE can alter the expression of SP1-target genes, such as *MAP1LC3B*.

### SopE contributes to down-regulating autophagy MAP1LC3B expression

**Figure 3:**
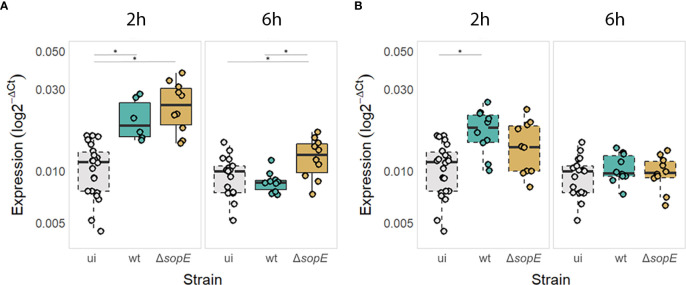
*MAP1LC3B* gene expression is increased at the early stages of *Salmonella* infection but decreased at later stages in a SopE-dependent manner in *Salmonella-*containing epithelial cells **(A)** but not in bystander cells **(B)**. *MAP1LC3B* expression levels expressed as log2-ΔCT so that uninfected cells (ui) are also displayed. Continuous borders = infected cells containing *Salmonella* (wt or mutant) and dashed borders = bystander epithelial cells not containing *Salmonella* or uninfected cells. * p<0.05.

SopE was shown to be essential in the SCV formation during invasion and it was also shown that some of the intracellular SopE protein remains associated with the SCV membrane at later time point during infection at a time when autophagy process is induced (Schlumberger and Hardt, 2005; Vonaesch et al., 2014). We first tested the functional importance of the direct interaction of SopE with SP1 by monitoring whether SopE influences the expression of the key *MAP1LC3B* gene, directly downstream of the SP1 transcription factor. For that, we infected HT-29 epithelial cell monolayers with either a *sopE^+^* strain of *S.* Typhimurium (=wt), or its Δ*sopE* deletion strain derivative. Both strains contain a ɸ(*ssaG’-gfp^+^*) transcriptional fusion, allowing intracellular GFP^+^ *Salmonella* containing HT-29 cells identifiable in a pool of infected and non-infected cells. At 2 and 6h p.i., *MAP1LC3B* RNA levels were quantified in *Salmonella*-containing cells or in epithelial cells that did not contain *Salmonella* despite being part of the infected cells (from then on called “bystanders”). *MAP1LC3B* RNA levels revealed that SopE impacts on the transcriptional levels of *MAP1LC3B* compared with non-infected cells (**Figure 3**), consistent with this pathogen-derived protein interacting with SP1 transcription factor (**Figure 2**).

Surprisingly, although SopE is essential for the early internalisation of *Salmonella* inside epithelial cells, it is not required for upregulating autophagy early during infection, e.g. 2h p.i. (**Figure 3A**). Epithelial cells upregulated *MAP1LC3B* expression irrespective of the presence or absence of SopE. Interestingly, *MAP1LC3B* gene expression was upregulated significantly even in bystander epithelial cells, suggesting that either the bystander cells respond to *Salmonella*-derived compounds that are sensed when the pathogen is not internalised or that they respond to mediators produced by the *Salmonella*-containing epithelial cells directly (**Figure 3B**).

However, at a later time point, when *Salmonella* started proliferating intracellularly (6h p.i.), we observed that the level of *MAP1LC3B* expression returned to that of non-infected cells in a SopE-dependent manner. This was particularly the case in HT-29 cells that contained *Salmonella* and not so for bystander epithelial cells that did not contain *Salmonella* cells (**Figure 3B**).

### SopE also modulates autophagy flux

As well as modulating the regulation of core autophagy gene expression through specific transcription factors, we hypothesise that SopE could also influence the autophagy flux as it is retained at the SCV membrane site several hours after *Salmonella* internalisation. MAP1LC3, once lipidated and recruited to the membrane nucleation site, connects the cargo to the vesicle membrane through the SQSTM1/p62 adaptor protein. We chose to follow MAP1LC3 and SQSTM1/p62 associated with the autophagosome as these two proteins are good indicators of the autophagy flux taking place in a mammalian cell (Xu et al., 2018). To address this, LC3 number of puncta and p62 intensity were assessed by immunofluorescence confocal bioimaging 6h p.i., at a similar time when SopE is still retained at the SCV membrane (See Materials & Methods section).

**Figure 4:**
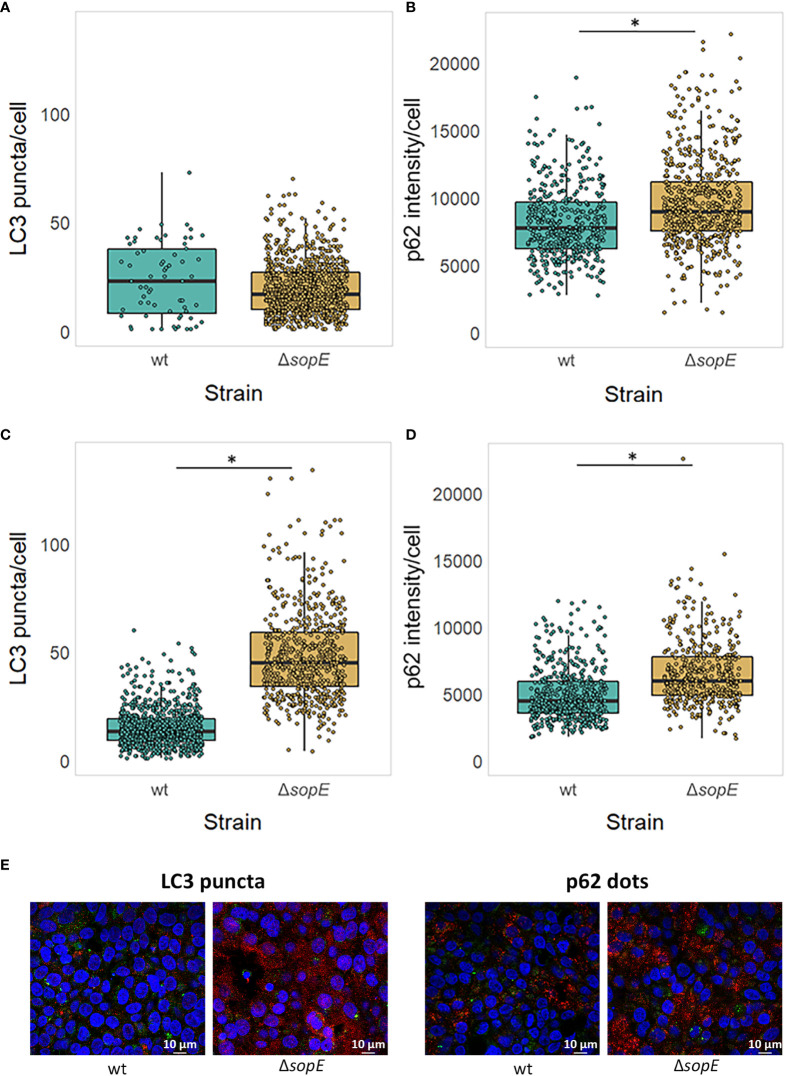
SopE dampens the autophagic flux by 6h post infection. LC3 puncta number and p62 dot intensity in HT-29 epithelial cells infected for 6h with either wt *Salmonella* strain or its Δ*sopE* gene deletion derivative. **(A, B)** HT-29 epithelial cells were infected with wt *Salmonella* and its Δ*sopE* gene deletion derivative strain as indicated before. **(C, D)** HT-29 cells were pre-treated with Rapamycin for 11h prior to and during the 6h-long infection (Maximum 17h). LC3 puncta **(A, C)** and p62 dot intensity **(B, D)** was quantified from HT-29 cells containing *Salmonella*. *p=0.05. **(E)** Micrographs of HT-29 cells pre-treated with Rapamycin showing LC3 puncta (red, left) and p62 dots (red, right), intracellular *Salmonella* cells (green) and nuclei (blue) illustrating panels **(C, D)**.

When autophagy is not pre-induced before infection by *Salmonella*, no significant difference in the number of LC3 puncta is observed at 6h p.i. (**Figure 4A**). However, the p62 dots intensity is significantly greater in cells infected with *Salmonella* that lack *sopE* (**Figure 4B**). This suggests that SopE, although dampening the expression of autophagy core genes at 6h p.i., simultaneously modulates the autophagy flux locally, at intracellular autophagy sites.

Whether SopE acts on the autophagy flux, before or after that process is induced, is not yet clear. LC3 puncta number and p62 dots intensity were therefore monitored also after autophagy was experimentally triggered. **Figures 4C**, **D** clearly show that both LC3 puncta (C) and p62 dot intensity (D) increase significantly when SopE is absent, confirming that the dampening effect SopE normally has on autophagy by the time *Salmonella* starts to replicate intracellularly. SopE’s effect is even amplified when autophagy is already activated.

### SopE modulates the autophagy flux only when Salmonella is within SCV and not when it has escaped the vacuole and is cytosolic.

Finally, we questioned whether the fraction of SopE retained at the SCV membrane implies that *Salmonella* benefits from the SopE-dampening autophagy only when residing within an SCV.

**Figure 5:**
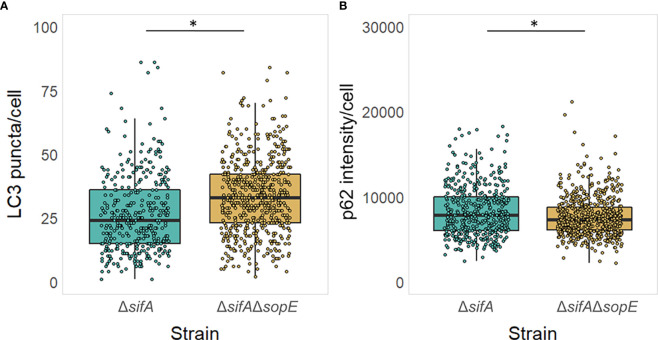
Cytosolic *Salmonella* no longer can dampen the autophagy flux in a SopE-dependent manner. **(A, B)** Autophagy was pre-induced with Rapamycin in HT-29 epithelial cell monolayers and maintained during the 6h infection with *Salmonella*, as it exacerbates the impact of SopE on modulating autophagy, making it easier to visualise. LC3 puncta **(A)** and p62 intensity **(B)** was quantified from HT-29 cells containing *Salmonella*. *p=0.05.

To address the impact of *Salmonella* intracellular localisation on the role of SopE as modulator of the autophagy flux, we constructed a strain lacking SopE that would essentially be located in the epithelial cell cytosol. SifA effector protein of *Salmonella* is well known for being instrumental to the evolution and maintenance of the SCV (Beuzón et al., 2002), recruiting vacuolar ATPase to the SCV, permitting the SCV luminal environment to acidify (Beuzón et al., 1999; Martin-Orozco et al., 2006), and the formation and extension in certain host cells to *Salmonella*-induced Tubules (SITs) from the endosomal system for the intracellular survival of *Salmonella* in the host (Liss and Hensel, 2015). It was shown in diverse studies that *Salmonella* strains lacking SifA escape the SCV and start proliferating anarchically in the cytosol of non-phagocytic cells such as epithelial cells. We therefore constructed the strain TK0026 carrying the double Δ*sifA* and Δ*sopE* genes deletion (See Materials & Methods). Similarly done to the experiment shown on **Figure 4**, HT-29 epithelial cell monolayers were infected this time with either the strain lacking SifA only or the strain lacking both SifA and SopE. **Figure 5** shows a mild, yet significantly different increase in the number of LC3 puncta in cells infected with *Salmonella* lacking SifA and SopE compared with the cells infected with *Salmonella* lacking SifA only, suggesting SopE could mildly influence MAP1LC3B lipidation and recruitment to the autophagosome membrane. However, the autophagy flux does not seem to be differing whether SopE is present or not once *Salmonella* is cytosolic, emphasising the specificity of SopE’s modulatory effect.

## Discussion

Our study predicts that several *Salmonella* virulence effectors proteins interact directly with some transcription factors involved in regulating autophagy, impacting the subsequent expression of core autophagy genes, such as *MAP1LC3B*. We also observed that *Salmonella* influences the autophagy flux at certain stages of infection; it does so in epithelial cells where *Salmonella* is still associated with the SCV. We had previously shown through protein-protein interaction network analysis that autophagy preferentially targets virulence effectors secreted by intracellular pathogens, and that pathogens have evolved mechanisms that conversely compromise the autophagy process. Autophagy-associated proteins are directly targeted by pathogens at different stages of the process (Sudhakar et al., 2019). Here we asked whether *S*. Typhimurium virulence effector proteins can influence this clearing process by acting on the regulatory level above the core autophagy genes, i.e. on the transcription factors that normally regulate them. Using protein-protein interaction predictions and transcription factor-gene interaction databases we built a network that links several *S*. Typhimurium virulence effectors to key transcription factors and predicted that *S.* Typhimurium can modulate the regulation of autophagy core gene expression. We showed that six SPI-1 effectors could interact with overall 33 transcription factors that normally regulate the expression of core autophagy genes (**Figure 1A**). As expected, SPI-2 SseL and SspH2 effectors showed a greater number of interactions with transcription factors, with 10 and all 33 regulatory targets, respectively. This reflects the adaptation of *Salmonella* to the host cell intracellular environment where SPI-2 secreted effectors are the main contributors to *Salmonella* survival and virulence (Haraga et al., 2008; Srikanth et al., 2011). Yet our study also highlighted new autophagy interactors among the *Salmonella* effector arsenal; for example, we showed that the *Salmonella* SseI [also called SrfH; (Thornbrough and Worley, 2012)] cysteine hydrolase can interact with 9 transcription factors modulating autophagy gene expression in epithelial cells. This effector might play an important role later in the infection, affecting the migration of macrophages and DCs (Jennings et al., 2017) although a single nucleotide polymorphism in the *sseI* gene that occurs naturally in some *Salmonella* strains prevents SseI from stimulating monocyte migration (Thornbrough and Worley, 2012). Our study suggests that SseI essentially impacts autophagy through its interaction with SP1 and TP53 transcription factors. We can envisage that, in addition to its regulatory role, it could also interfere with proteolytic processes taking place within epithelial cells through its cysteine hydrolase activity; initiation, execution, or inhibition of the autophagy process being highly dependent on proteases activity (Kaminskyy and Zhivotovsky, 2012).

Fewer SPI-1 effectors seem to interact with autophagy regulators (**Figure 1A**). This is in agreement with SPI-1 effectors being mostly involved in the entry of the pathogen into non-phagocytic mammalian cells (Lou et al., 2019). However, certain SPI-1 effectors have been shown to persist within the host cells, even past the point when *Salmonella* starts replicating. For instance, the SPI1 SlrP E3 ligase can also bind to a large number of transcription factors (**Figure 1A**) highlighting the importance of interfering with the ubiquitin pathway of the host, and possibly assisting the interaction of E3 ligases normally secreted through SPI-2 T3SS (e.g. SspH2; (Herhaus and Dikic, 2018).

The two *Salmonella* SPI-1 GEFs-mimicking effectors SopE and SopE2 seem to have a more specific impact on autophagy gene expression as they can interact with much fewer transcription factors (**Figures 1A**, **B**). SopE and SopE2 effectors can both target two transcription factors, TP53 and SP1, affecting autophagy induction through ULK1 and FIP200, respectively, and the autophagosome formation through SQSTM1/p62, UVRAG and MAP1LC3B, rather than the late lysosome-mediated clearance of the cargo. This restricted number of transcription factors potentially interacting with SopE and SopE2, as shown here for SP1 and SopE, suggests a very specific role of these effectors in the hijacking of autophagy by *Salmonella*. TP53 was also predicted to be a binding target of SopE and, although the role of this interaction was not studied here, its role in *Salmonella*-mediated autophagy gene expression regulation should be further explored. Indeed, when the transcription factor TP53 is inhibited or absent, increased autophagosome formation and overall autophagy flux is observed, suggesting that cytosolic TP53 reduces autophagy initiation. TP53 transcription factor acts upon autophagy modulation even when *Salmonella* remains cytosolic (Tasdemir et al., 2008; Kroemer et al., 2015). We suggest that SopE inhibits autophagy induction through its binding to TP53, maintaining this transcription factor in the cytosol and exacerbating its autophagy dampening effect. *sopE2* is present in all pathogenic strains of *Salmonella*, while *sopE* is assumed to have appeared later in the *Salmonella* evolution by duplication of *sopE2* and is present only in a subset of strains such as SL1344 used in this study (**Table 1**) (Bakshi et al., 2000; Mirold et al., 2001), suggesting a gained function in these strains. Indeed, SopE was also obtained from the strain responsible for a major epidemic in the 1970s and 1980s (Mirold et al., 1999). We focussed particularly on SopE to study its added impact on core autophagy gene expression and autophagy flux modulation by these *Salmonella* strains.

We first validated experimentally that SopE can bind to the SP1 transcription factor regulating MAP1LC3B expression (**Figure 2**), validating our prediction (**Figure 1B**). We then showed that, following the early increase in autophagy associated with the infection by *Salmonella* (2h p.i.), SopE contributes to the dampening of *MAP1LC3B* gene expression (**Figure 3**), and of the autophagy flux (**Figure 4**) in the HT-29 cell line. Indeed, at 6h p.i., this effect observed with the wildtype strain was no longer observed when SopE was missing. Acting on key transcription factors directly is likely to be an evolutionary selected mechanism for *Salmonella* to control host clearance function. We propose that SopE induces autophagosome formation through SP1. Our regulatory network analysis showed that SP1 transcription factor regulates several core autophagy genes, including *MAP1LC3B* and p62/*SQSTM1* (**Figures 1A, B**). SP1 has also been shown to repress the autophagy process in malignant epithelial cells by dampening *p62* expression (Xu et al., 2018). Here, we show that SopE dampens the autophagy flux. Indeed, accumulation of p62 protein is a commonly used indication for autophagy impairment or decrease. Here, we observed a decrease in p62/SQSTM1 protein associated with autophagosomes that depended on SopE being present (**Figures 4C, D, E**). We suggest that SopE, through its interaction with the SP1 transcription factor, negatively modulates the autophagy flux, protecting vacuolar *Salmonella* from lysosomal degradation.

We observed however that the effect of SopE in dampening autophagy only happens when *Salmonella* is associated with the SCV. Conversely, when *Salmonella* was mostly cytosolic due to the deletion of *sifA*, SopE no longer seemed to have the striking effect on autophagy dampening we saw when *Salmonella* remained mostly associated with the SCV compartment (**Figures 4** vs **5**). A previous study showed that infection-induced autophagy also contributes to the healing of damaged SCV by SPI1 proteins secreted through theT3SS, helping the endosomal/SCV maturation (Kreibich et al., 2015). This benefits consequently intravacuolar *Salmonella* cells, as these can proceed with SPI-2 effector-mediated intracellular survival and proliferation. In our study, the SopE protein no longer dampens autophagy flux in host cells where *Salmonella* has escaped the vacuolar compartment or resides within damaged SCV that will require part of the autophagy flux to repair the endosomal/SCV membrane. This process could take place in parallel to epithelial cell compartment size regulation which determines whether the pathogens will escape the vacuole or trigger enlargement of the vacuole by fusion with infection-associated macropinosomes as previously described (Stévenin et al., 2019).

The prediction that both SopE and SopE2 effectors can similarly affect autophagy highlights even more the importance of these molecular mimics across a broad spectrum of *S.* Typhimurium strains. In strains expressing both *sopE* and *sopE2* genes, modulation of autophagy induction and autophagosome formation might play a key role in strongly hijacking host functions that respond to autophagy modulation, such as antimicrobial production, possibly to help maintaining intestinal *Salmonella* populations and ensure spreading of the pathogen. However, as harbouring the evolutionarily newer *sopE* suggests a gained function in these strains compared to having *sopE2*, it is crucial to do follow up experiments confirming or contradicting the predictions.

Our experimental strategy to physically separate bystanders from epithelial cells that contain *Salmonella* revealed clear differences between these two categories of host cells as well as between bystanders and uninfected cells. Indeed, bystanders seem to participate in the host response to *Salmonella* infection as already shown for other pathogens such as *Shigella flexneri*, where bystanders were responding to infection in an effector-independent manner (Lippmann et al., 2015). Cross-talk between infected and uninfected neighbouring cells has previously been described for many bacterial or viral pathogens, for example through cytokines signalling (Milivojevic et al., 2017; Bost et al., 2020), or directly through uptake of pathogen effector proteins from infected to uninfected cells (Guidi et al., 2013). *S*. Typhimurium was also shown to cause a reprogramming of microRNAs in infected cells, affecting also bystander cells (Aguilar et al., 2021). Endoplasmic reticulum stress response is activated in bystander cells, affecting many genes’ expression; in particular downregulating key transcription factors, such as E2F1. E2F1 normally activates autophagy and was shown to work in synergy with the SP1 transcription factor (Lin et al., 1996) propose that SP1 is one target of the *Salmonella*-derived SopE GEF-mimicking protein, affecting autophagy regulation, possibly in combination with E2F1 down-regulation, although to a lesser degree in bystanders cells. This is in agreement with SopE acting locally within infected cells without affecting the surrounding cells, possibly as a hiding mechanism from host innate defense. It is also possible that bystanders would follow the same pattern at a later time point if there is a delay in their response to what is happening in *Salmonella*-containing cells.

The HT-29 cell line we used is a cancer-derived cell line, and like many lines it carries several mutations that affect the normal functioning of the cells. However, along with other cell lines used to study *Salmonella*-host cell interactions, HT-29 cells carry no mutation in core-autophagy genes (unpublished data). Cancer cell lines present the advantage to study the pathogen’s behaviour in a very homogenous host cell population, yet they only partially mirror what is happening in native tissue. With the growing evidence of how useful intestinal organoid-based models are to study host-microbe interactions, including *Salmonella*-host interactions, using them for validation of network-based predictions of how pathogens’ effectors interfere with host cellular pathways is the obvious next step to pursue.

Overall, our study emphasises the power of network analysis approach in identifying potential interactions between pathogen effector proteins and host cellular machinery regulation. We had previously demonstrated that protein-protein interactions between secreted pathogen effectors and core autophagy proteins is a conserved strategy for many intracellular pathogens (including *Salmonella*) to modulate the host autophagic clearance mechanisms (Sudhakar et al., 2019). Here, using a similar approach, we showed that *Salmonella* targets also the regulation of those core autophagy genes. Our experimental validation emphasises the possible role of SopE effector protein as such a local regulator of autophagy flux. Future complementary work investigating the mechanism behind SopE/SP1 or SopE/TP53 interactions will add to our understanding of the complexity and fine tuning of how host-pathogens crosstalk has developed.

## Data availability statement

The list of Salmonella effectors, relevant host transcription factors and their first neighbours used in the study have is available as supplementary data. The scripts used in the study are available at: https://github.com/korcsmarosgroup/HMIpipeline.

## Author contributions

AD has conducted the majority of the computational and experimental work. She contributed to the data analysis as well as the writing and editing of the manuscript. A-CJ has performed all microscopy work, image analysis for this study and the writing of the relevant methods section. LG has contributed to the computational work of this study. IM, JL and AL have contributed to the Flow cytometry work, RNA extraction from low input samples and the writing of the corresponding method section. PB, RI and RK have generated the initial Salmonella sopE gene deletion strain used in this study, and to the writing of the manuscript. IN has contributed to the writing and editing of the manuscript. TK contributed to the conception of the study and the writing and editing of this manuscript. Finally, IH contributed to the conception of the study, to some experimental work, to the data analysis. She led the writing and editing of the manuscript. All authors contributed to the article and approved the submitted version.

## Acknowledgments

We are truly grateful to Jost Enninga (Department of Cell Biology & Infection, Pasteur Institute, Paris, France) for his instrumental advice and discussion. The work of AD, RI, TK and IH were supported by the UKRI BBSRC Gut Microbes and Health Institute Strategic Programme BB/R012490/1 and its constituent projects BBS/E/F/000PR10353 and BBS/E/F/000PR10355 as well as a BBSRC Core Strategic Programme Grant for Genomes to Food Security (BB/CSP1720/1) and its constituent work packages, BBS/E/T/000PR9819 and BBS/E/T/000PR9817. LG ​​was supported by a BBSRC - Norwich Research Park Biosciences Doctoral Training Partnership grant (BB/M011216/1). RK was supported by research grants BB/N007964/1 and BB/M025489/1, and by the BBSRC Institute Strategic Programme Microbes in the Food Chain BB/R012504/1 and its constituent projects BBS/E/F/000PR10348 and BBS/E/F/000PR10349. ACJ and IPN were supported by BBSRC grants BB/L006324/1 and BB/P007856/1.

## Conflict of interest

The authors declare that the research was conducted in the absence of any commercial or financial relationships that could be construed as a potential conflict of interest.

## Publisher’s note

All claims expressed in this article are solely those of the authors and do not necessarily represent those of their affiliated organizations, or those of the publisher, the editors and the reviewers. Any product that may be evaluated in this article, or claim that may be made by its manufacturer, is not guaranteed or endorsed by the publisher.
